# Living With, Managing and Minimising Treatment Burden in Long Term Conditions: A Systematic Review of Qualitative Research

**DOI:** 10.1371/journal.pone.0125457

**Published:** 2015-05-29

**Authors:** Sara Demain, Ana-Carolina Gonçalves, Carlos Areia, Rúben Oliveira, Ana Jorge Marcos, Alda Marques, Ranj Parmar, Katherine Hunt

**Affiliations:** 1 Faculty of Health Sciences, University of Southampton, Southampton, SO17 1BJ, United Kingdom; 2 NIHR Wessex Collaboration for Leadership and Research in Health Care, Southampton, United Kingdom; 3 School of Health Sciences, University of Aveiro (ESSUA), Agras do Crasto—Campus Universitário de Santiago, Edifício 30, 3810–193 Aveiro, Portugal; 4 Unidade de Investigação e Formação sobre Adultos e Idosos (UNIFAI), Porto, Portugal; Supportive care, Early DIagnosis and Advanced disease (SEDA) research group, UNITED KINGDOM

## Abstract

**Background:**

‘Treatment burden’, defined as both the workload **and** impact of treatment regimens on function and well-being, has been associated with poor adherence and unfavourable outcomes. Previous research focused on treatment workload but our understanding of treatment impact is limited. This research aimed to systematically review qualitative research to identify: 1) what are the treatment generated disruptions experienced by patients across all chronic conditions and treatments? 2) what strategies do patients employ to minimise these treatment generated disruptions?

**Methods and Findings:**

The search strategy centred on: treatment burden and qualitative methods. Medline, CINAHL, Embase, and PsychINFO were searched electronically from inception to Dec 2013. No language limitations were set. Teams of two reviewers independently conducted paper screening, data extraction, and data analysis. Data were analysed using framework synthesis informed by Cumulative Complexity Model. Eleven papers reporting data from 294 patients, across a range of conditions, age groups and nationalities were included. Treatment burdens were experienced as a series of disruptions: biographical disruptions involved loss of freedom and independence, restriction of meaningful activities, negative emotions and stigma; relational disruptions included strained family and social relationships and feeling isolated; and, biological disruptions involved physical side-effects. Patients employed “adaptive treatment work” and “rationalised non-adherence” to minimise treatment disruptions. Rationalised non-adherence was sanctioned by health professionals at end of life; at other times it was a “secret-act” which generated feelings of guilt and impacted on family and clinical relationships.

**Conclusions:**

Treatments generate negative emotions and physical side effects, strain relationships and affect identity. Patients minimise these disruptions through additional adaptive work and/or by non-adherence. This affects physical outcomes and care relationships. There is a need for clinicians to engage with patients in honest conversations about treatment disruptions and the ‘adhere-ability’ of recommended regimens. Patient-centred practice requires management plans which optimise outcomes and minimise disruptions.

## Introduction

Living with and managing chronic illness requires hard work as patients seek to cope with, adapt to and minimise the physical, emotional and biographical impacts of the disease [1, 2]. Navigating services, interacting with health professionals and enacting treatments also creates work and may generate disruptions to patients’ wellbeing and functioning [3].This has been termed treatment burden or burden of treatment (BoT).

Conceptual clarity is vital in research and practice. Careful delineation of the causes, components and consequences of BoT will enhance attempts to ameliorate it; however, BoT is an emergent concept which researchers are still working to define. Some have conceptualised treatment burden as the physiological side-effects (e.g. pain, nausea, dizziness, rash) of medication, surgery or other therapies [4–6], whilst others have explored BoT from the perspective of psychosocial consequences [7] and reductions in quality of life [8–12]. Yet others have focussed on the workload arising from treatment regimens [12], conceptualising treatment burden as “the self-care practices that patients with chronic disease must perform to enact management strategies and respond to the demands of healthcare providers and systems”. Treatment work load is situationally specific; the nature of work and its associated burdens vary in different countries, partly attributable to differences in the structure and funding of healthcare systems [3]. The focus on ‘treatment workload’ has usefully led to the application of Normalisation Process Theory (NPT) [13] as an analytical framework and the development of a taxonomy of physical, cognitive and interactional tasks contributing to treatment burden [11]. Further research, using qualitative data, has defined treatment burden as both the workload of treatments **and** their impact on “patient functioning and well-being” [3]. Analysing qualitative interviews (n = 32), across a range of conditions, Eton et al identified the “work patients must do”, “the strategies and tools which facilitate self-care” and the “factors that exacerbate burden”. Sav, King [14] conducted a concept analysis of treatment burden in a range of chronic illnesses. They described the “dynamic and multidimensional” attributes of BoT which consisted of “both subjective and objective elements” and highlighted the need for a focus beyond workload. A series of antecedents (e.g. ‘patient characteristics’ and ‘health care systems’) and consequences (e.g. ‘adherence’, ‘resource use’) were also characterised. Whilst the complexity and fluidity of treatment burden were acknowledged, the conclusions were limited by the paucity of inductive, qualitative research exploring patient accounts included (n = 1 paper). Further research to describe and classify treatment generated disruptions is required.

In this research we set out to build and extend the body of work on conceptualising treatment burden, across all chronic conditions and treatments, by systematically reviewing empirical qualitative research to answer the following questions: 1) what are the treatment generated disruptions experienced by patients across all chronic conditions and treatments? 2) what strategies do patients employ to minimise these treatment generated disruptions?.

Table 1 summarises the PICOS rationale.

**Table 1:** PICOS table summarising study rationale.

**P**articipants	Humans, any age, any condition
**I**nterventions	Any treatment
**C**omparisons	Not applicable
**O**utcomes	Treatment burden or Burden of treatment
**S**tudy design	Qualitative data collection and qualitative analysis of patient perspectives

## Methods

### Search strategy

Qualitative studies using methods involving direct patient contact, such as interviews and focus groups, and seeking to understand the patient experience of treatment burden across all conditions and treatments were sought. Searching and screening were conducted according to the PRISMA statement (See S1 PRISMA Checklist) [15]. The data bases Medline, CINAHL, Embase, and PsychINFO were searched electronically. No date limitations were set but “language” was restricted to English or Portuguese as there were no resources for translation. We aimed to identify all papers that used the terms “treatment burden” or “burden of treatment” in their title or abstract. Given that the systematic identification of qualitative research is problematic [12, 16] we did not limit our initial search by research method. Rather, identification of qualitative papers was undertaken during the blinded screening process. Initial searches were conducted in June 2012 and were updated in April 2014.

### Data screening, extraction and analysis

Title, abstract and full paper screening were undertaken independently by three researchers (AJM; ACG; CA) using a data-extraction proforma designed and piloted by the team. Inclusion was accepted by concordance; a third party (SD or KH) resolved any disagreements. Duplicates and any papers not addressing treatment burden at the level of the patient were excluded (e.g. global economic treatment burden; treatment burden on services). Papers were included only if they utilised recognised inductive qualitative data collection **and** analysis methods. Quantitative research, systematic reviews, qualitative syntheses, opinion pieces and papers reporting qualitative methods but containing no qualitative data (e.g. quotations or thematic frameworks) were excluded.

Qualitative research is interpretative: data therefore included verbatim quotes and authors’ interpretative comments and were extracted from the findings/results and discussion sections of papers [11]. Data were analysed using framework synthesis [17, 18] using a coding framework informed by the Cumulative Complexity Model (CCM) [19]. The CCM proposes that the balance between patient workload (treatment, ‘everyday’ and occupational tasks) and their capacity to undertake that work influences access and adherence to treatments and consequent health outcomes. This model was appropriate to our focus on understanding how BoT impacts on “patient functioning and well-being” [3] and the factors contributing to and shaping these experiences. We used Shippee et al’s categories (e.g. capacity, workload, adherence etc.) to generate our framework but did not pre-determine the existence of their proposed inter-category relationships.

Framework synthesis uses a two staged approach; data extraction and management into pre-determined categories and then thematic analysis to identify patterns of data within and between categories [17]. A data management framework, with 4 well defined categories (workload, capacity, treatment impact and engagement/adherence) was developed. Data assignment was undertaken by pairs of researchers (SD/CA: papers 1–4; KH/RO: papers 5–8; SD/AM: papers 9–11) who coded blind and then met in pairs to discuss and agree categorisation. Finally, each pair presented their coding to the other and any issues of contention were discussed and agreed by the whole team.

The second phase of framework synthesis involved thematic analysis of data categorised within the ‘treatment impact’ category. Two researchers (SD/KH) conducted this analysis separately, and then through collaborative discussion, using paper based labelling, fragmenting, comparing and grouping [20] until a clear taxonomy of the components of treatment impact were agreed. Finally, relationships between the treatment impacts and those proposed previously (e.g. capacity, workload and adherence) were identified using constant comparison, team brainstorming and diagrammatic modelling. Our synthesis generated both second-order (interpretations offered by the original researchers) and third-order constructs (new interpretations beyond those offered in individual studies) [21].

Our emerging synthesis indicated that people seek healthcare not simply to relieve physical or emotional symptoms but because those symptoms stop them from doing what they want (e.g. running or hiking) and being who they want to be (a professional athlete, a member of the rambling club or an optimistic person). In finalising our synthesis model we therefore called upon Sen’s capability approach [22] which considers the genuine opportunities (capabilities) people have to achieve the kind of lives they value: to feel like, do what and be who they want to be. Treatment burdens were therefore considered to be any treatment generated disruption in people’s ability to feel, do or be who they wanted to be.

### Quality appraisal

Quality appraisal was undertaken using the Critical Appraisal Skills Programme [23] criteria for qualitative studies. Quality appraisal was independently conducted by two researchers (SD and KH) and answers compared and discussed. Studies were not, however, excluded on the basis of quality.

### Enhancing analytic credibility through expert patient review

Analytic relevance and credibility was enhanced by inclusion of a lay representative in the study team (RP). RP brought the following expertise to the team: i) experience of a long-term condition (stroke); ii) pre-stroke expertise in analysing complex systems and processes iii) leadership of local and national patient organisations. RP critiqued and challenged the emergent analysis, reflecting on its relevance to his own experience and those of other people with long-term conditions that he worked with.

## Results

### Retrieved studies

**Fig 1:**
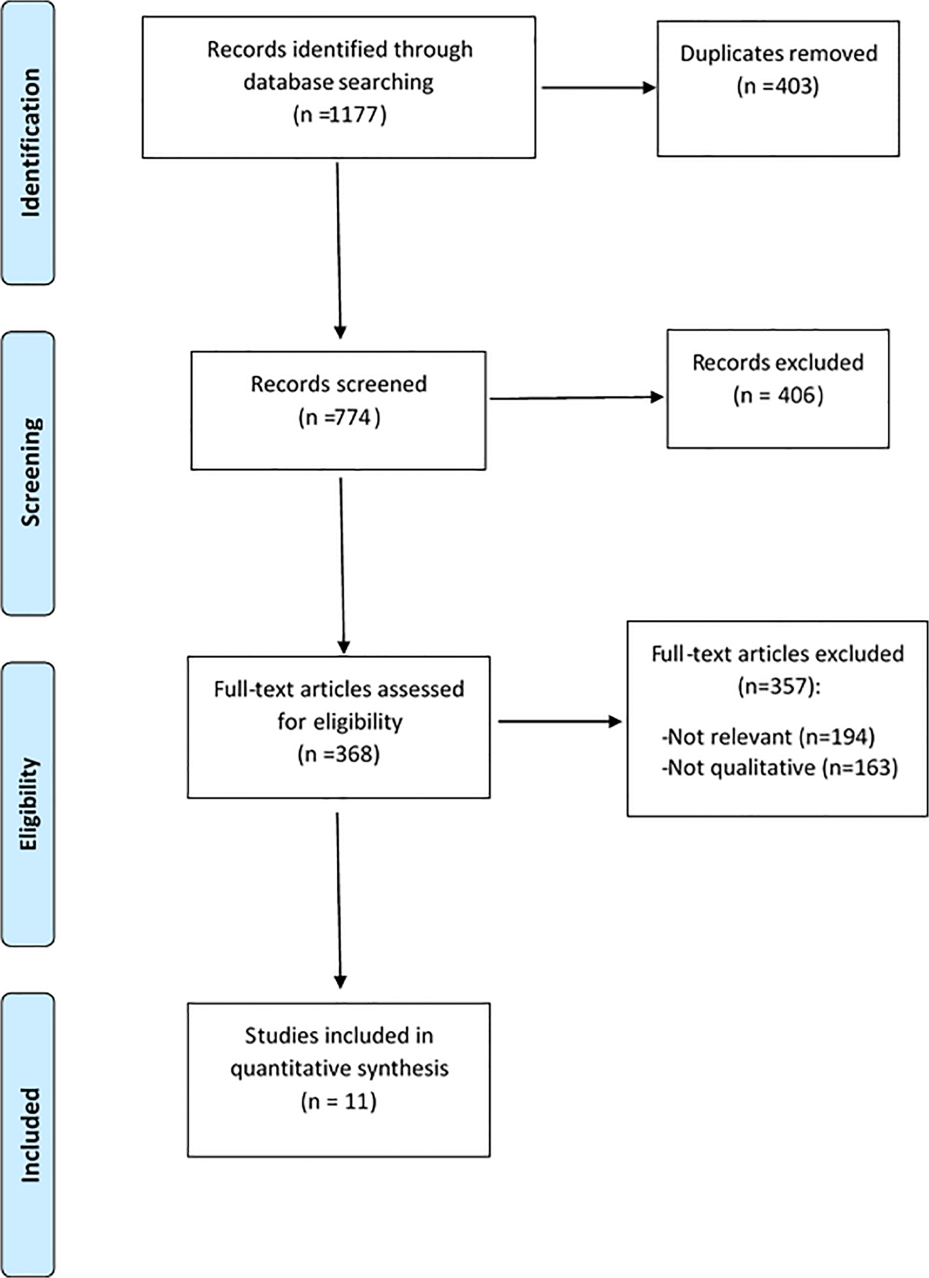
PRISMA flow diagram indicating inclusion and exclusion criteria of papers at each stage of screening.

The initial (April 2012) and updated (April 2014) searches identified a total 1177 papers; after removal of duplicates, 774 titles and abstracts were screened for relevance; 368 full text articles were assessed for eligibility; 11 papers which used qualitative methods **and** analysis and which presented data on patients’ perspectives of treatment burden were included. Fig 1 presents the PRISMA flow diagram indicating the inclusion and exclusion of papers at each stage of the screening process.

### Study details

Details of included studies are presented in Table 2. A range of qualitative methods were reported: ten used semi-structured interviews, either alone [3, 7, 24–28], with focus groups [29, 30] or with structured measures [8]; one conducted secondary analysis of existing qualitative data [11]. Included papers addressed a range of chronic conditions across the life-course (aged 8–96 years) including: adults with spasmodic dysphonia [7]; chronic heart failure (CHF) [11]; conditions requiring percutaneous endoscopic gastrostomies (PEG) [8]; chronic kidney disease (CKD) [24, 28]; people with limited life expectancy due to various conditions [29]; adolescents/young adults with cystic fibrosis (CF) [25] and tuberculosis (TB)[30]; children with Primary Ciliary Dsykinesia (PCD)[26] and, a range of chronic conditions [3, 14]. The conditions varied in terms of the severity and impact of disease, the likelihood and immediacy of life threat, and the invasiveness and criticality of treatments. Studies were undertaken in: the UK [8, 11, 24, 26], US [3, 7, 25, 29], Nepal [30], Australia [27], and Greece[28]. All of the papers included a mix of genders. A variety of qualitative analysis methods were used; all sought to identify common themes raised by participants.

**Table 2:** Details of included papers.

Authors	Study reference	Year	Country	Study design	Participants N (ages)	Condition(s) Studied	Stated Focus
**Eton et al**	3	2012	USA	Interviews	32 (26–85 years)	Complex patients with chronic diseases and polypharmacy	Burden of treatment from the perspective of the complex patient
**Baylor et al**	7	2007	USA	Interviews	6 (49–80 years range)	Spasmodic Dysphonia	Psychosocial consequences of treatment
**Jordan et al**	8	2006	UK	Interviews + structured rating scale and QoL measure	20 (24–84 years range)	Long-term percutaneous endoscopic gastrostomies	Burden of treatment from a patient perspective
**Gallacher et al**	11	2011	UK	Secondary analysis of qualitative interview	47 (45–88 years range)	Chronic Heart Failure	Patients’ experiences of treatment burden
**Johnston and Noble**	24	2012	UK	Interviews	9 (74–96 years range)	Chronic kidney disease	Burden of treatment and impact on treatment choice
**George et al**	25	2010	USA	Interviews	25 (16–35 years range)	Cystic Fibrosis	Barriers and facilitators to treatment adherence
**Schofield and Horobin**	26	2014	UK	Interviews	5.(8–15)	Primary Ciliary Dyskinesia	Physiotherapy treatment experiences
**Sav et al**	27	2013	Australia	Interviews	97 (16–83; mean 57.2)	Chronic conditions	Treatment burden
**Karamandiou et al**	28	2013	Greece	Interviews	7 (32–68 years)	End Stage Renal Disease	Illness beliefs, treatment experiences and adherence
**Fried and Bradley**	29	2003	USA	Focus groups and interviews	23 (mean age of 70 years)	Congestive heart failure, chronic obstructive pulmonary disease, or cancer with limited life expectancy	End-of-Life treatment decisions
**Lewis and Newell**	30	2009	Nepal	Interviews and Focus groups	23 (age not stated)	Tuberculosis	Improving care and understanding patient support

### Quality appraisal

Papers were moderate to high quality. The sampling strategy, relationship between researchers and participants and detailed consideration of ethical issues were the weaker elements of these papers.

### Thematic findings

**Fig 2:**
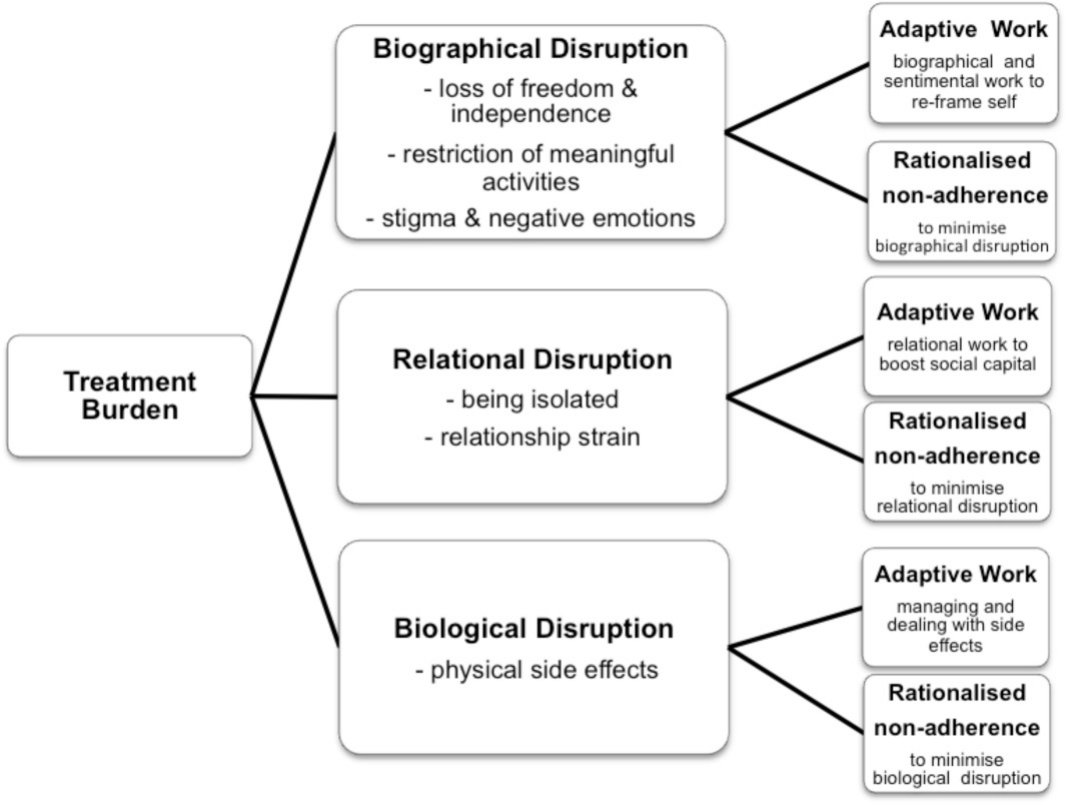
The biographical, relational and biological disruptions generated by treatment burdens and the strategies of adaptive work and rationalised non-adherence which patients employ to minimise these.

The synthesis generated eight second-order constructs related to the “negative impacts of treatment on functioning and well-being” which we collated into three third-order constructs “biographical, relational and biological treatment disruptions”. Table 3 presents these 2^nd^ and 3^rd^ order constructs and identifies where evidence for each can be found. The synthesis generated a further two third-order constructs related to the strategies employed by patients to minimise the disruptions to their valued capabilities: “adaptive treatment work” and “rationalised non-adherence”. This is also presented graphically in Fig 2.

**Table 3:** Definitions of the 2nd and 3rd order constructs identified, how they inter-relate and where evidence for each can be found.

How treatment burden was experienced (2nd-order constructs)	Definition/description	Papers with data on this theme	Capability disruptions	Response to capability disruptions (3rd-order constructs)
Physical symptoms and side effects	Negative impacts of treatments on body functioning experienced by patients in terms of treatment side-effects. For instance: pain, nausea, dizziness, breathlessness, fatigue, infection etc. This theme also included physical effects which occurred as a result of treatments or health service or interactional failures (e.g. pain when feeding tube blocked due to poor information from HCP)	3, 7, 8, 11, 24, 26, 27, 28	Biological disruption	Rationalised mal-adherence And/or Adaptive work: Managing and dealing with side effects of treatment
Negative Emotions	Negative affective states such as anxiety, fear, guilt, frustration which were experienced in anticipation of, during or as a consequence of treatments.	7, 8, 11, 24, 25, 27, 28, 29, 30	Biographical disruption	Rationalised mal-adherence And/or Adaptive work: Sentimental and biographical work to reframe self
Stigma and identity disruption	Negative changes in how patients perceived themselves or are perceived by others which arise as a consequence of treatment(s)	7, 8, 24, 25, 26, 27, 28, 29, 30		
Living with uncertainty	Unpredictable and unstable physical, psychological and social outcomes affecting people’s ability to plan and act in the short and/or long-term	7, 8, 11, 25, 27, 30		
Loss of freedom and independence	Feeling and being constrained by the requirements of enacting treatments and monitoring outcomes	7, 8, 24, 25, 26, 28, 29		
Loss or restriction of meaningful activities	Being unable to or restricted in performing valued occupational, leisure and family roles and activities by treatment actions or consequences.	7, 8, 11, 25, 28, 29, 30		
Feeling isolated and inadequately supported	Feeling alone whilst trying to cope with treatment activities or becoming isolated as a consequence of treatment.	7, 8, 11, 24, 27, 29, 30	Relational Disruption	Rationalised mal-adherence And/or Adaptive work: Relational work to sustain and repair relationships
Experiencing Relationship strain	Tensions in relationships with family and friends. These were caused by differing opinions about need for and quality of treatment adherence, reminders from families to engage in treatment or restrictions to the lives of family members	3, 26, 28		

#### Biographical disruption

The concept of biographical disruption was first defined by Bury [2] to explain the disruption to a person’s self-narrative and self-concept that results from chronic illness [31]. In our review, treatments were similarly identified as a cause of biographical disruptions impacting on people’s sense of self, negatively affecting their emotions, their sense of freedom and their ability to engage in meaningful activities. The majority of studies [7, 8, 24–28, 30] highlighted patients’ concerns about the impact treatments had on how they were seen by others and how they viewed themselves. Stigma was reported when enacting treatments (e.g. carrying a portable feeding system) or treatment consequences (altered voice following BOTOX) increased the visibility of otherwise hidden illnesses; when treatments involved others observing bodily sites, processes or excretions that were intimate or generated repulsion (e.g. sputum clearance; changing PEG tubes); or, when physical side-effects were embarrassing and impacted on identity (e.g. weak breathy voice post-botox):

*B*.*W*. *adjusts her work activities because of how people respond to her when she is in the breathy voice phase [post-botox]*: *“When you have the Marilyn Monroe voice*, *you don’t go into important situations*. *They just discredit what you say*. *Even my friends who are completely on my side [say] how can we take you seriously? It’s just too funny to listen to Marilyn Monroe [her identity with the breathy voice]*.*”* [7]

Loss of freedom and independence was a recurring theme in six of the reviewed papers [7, 8, 11, 25, 27, 30]. This included the practical loss of freedom conferred by virtue of the time taken to perform treatments and being physically constrained by technologies such as nebulisers, dialysis and feeding machines. Loss of freedom also incorporated the more existential sense of not being “carefree”.

The need to constantly plan treatments into daily regimes was viewed as loss of spontaneity, particularly amongst adolescents with CF: a time in the life-course normally associated with increased freedom and spontaneity. The need to undertake regular treatments and/or monitor treatment outcomes served as a constant reminder of being ill, even when symptoms had been eradicated.

*I used to take detailed notes (of my treatments and outcomes) and I thought later on “this is excessive”… When I was thinking about my voice all the time…it turned out to be more of a pressure thing*. [7]

The need to plan ahead was also linked to the problem of uncertainty [7, 8, 11, 26, 28–30] as unpredictable treatment outcomes and side-effects created the need for a life lived with contingency plans. Some uncertainty arose from the illness itself, however, some was directly attributable to the treatment. Causes of uncertainty included technological failures (e.g. feeding tubes becoming blocked), unpredictable responses to medication (botox), lack of easily observable treatment benefits (CHF, CF, TB), uncertainty about long term side-effects (CHF and botox), how to administer treatments (PEG feeding) or the purpose or duration of the regime (TB). Contradictory advice from health staff was also a major cause of uncertainty [28].

Negative emotional consequences were reported in all studies in this review. Emotional responses were highly variable and related to the individual patient and their social and treatment context. Patients responded with frustration or anger when they perceived treatment generated burdens to be avoidable e.g. when a lack of staff expertise or knowledge caused preventable complications or wasted patients’ time or when scheduling of treatments and appointments was hindered by inflexible services [3, 7, 8, 11, 24, 27, 30]. Anxiety, fear and worry were highlighted in several studies [3, 7, 8, 27, 28, 30]. People worried about the immediate and long-term risks of treatment, the future effectiveness of treatments, experiencing pain, losing employment, being stigmatised by others, the financial implications of treatment and becoming a burden to families. Guilt was experienced in relation to the physical workload or financial costs of treatment incurred by patients’ families and by patients who were unable to adhere to treatment recommendations; however, this could be exacerbated or ameliorated by the quality of relationship between patients and professionals.

*“[The doctor] was really funny and outgoing and really nice and that helped a lot getting me back into clinic*. *Because I don’t come as often as I should*, *but I come a lot more than I used to*.*”* [25]

The majority of studies [7, 8, 11, 25–27, 29] identified the reduction or loss of valued activities as a key element of biographical disruption. People receiving Botox injections weighed up when to have further injections based on the impact it would have on their valued activities. For instance one woman, who experienced breathlessness post-Botox, would not have the injection in the summer when she liked to go hiking, whilst another tried to plan the injection to avoid Christmas and other critical time points. People with CHF also reported avoiding travelling if they had taken diuretics or avoided diuretics if they wanted to travel, and adolescents with CF made similar decisions modifying their time-consuming treatment regimes so that they could do the things they wanted.

*Holding down a full time job and living life normally—time is a big thing…I would rather do all the things I want to do instead of sit home and do all the things I should and miss out on a bunch of stuff… I am quality over quantity*. [25]

Some treatments were not easily modified, for instance people undergoing PEG feeding or haemodialysis could not stop or reduce their treatments without major consequences. However, substantial restrictions to important activities were often deemed acceptable because the treatment was “life-saving”.

*It (PEG) dominates life—it’s in use the greater proportion of the 24 hours*. *It dominates movement*, *but we’re happy with it—if it wasn’t for the PEG she wouldn’t be here*.*’* [8]

#### Relational disruption

The negative impact of treatments on valued relationships was another common theme in the reviewed studies.

Treatments generated feelings of isolation: for instance, people with TB hospitalised far from family and friends; others had restrictive home-based regimes (PEG feeding, dialysis); and, children with PCD reported deliberately isolating themselves from friends to avoid the stigma of expectorating sputum. People requiring highly specialist treatments (e.g. PEG feeding and Botox) described isolation from appropriate professional support as a consequence of limited specialists in their community. This resulted in professional uncertainty about appropriate responses to complications, a lack of guidance about treatments and consequently, some patients experienced additional physical side-effects, emotional distress, and uncertainty. Potentially avoidable burdens were less well tolerated than those viewed as inherent to the treatment.

*When he first had the PEG they (nurses on the ward) pulled the curtains round the bed and called me back*. *They told me*, *‘You’ll be doing all this tomorrow’*. *There was just this one short instruction on the last night in hospital*. *He came home and nobody came to help us*. *The district nurse had gone sick*. *And we had the baby in the house as well*. *My daughter had just gone into hospital with the afterbirth retained—she was in two weeks*. *The baby was two weeks old*. *There was no help at all*. *You just had to manage*. [8]

Parents reported the strain that could arise in relationships with children [26]. Children with PCD often had differing opinions from their families on the quality and frequency with which they engaged in their physiotherapy and nebuliser regimes. Some parents suggested their children were “lazy” and needed “nagging” which created tension in the relationship and emotional impacts for both parents and children.

Relationship strain was also evident when time or financial resources spent on treatment had a negative impact on family leisure activities [26, 28] and some seriously ill people declined labour intensive treatments for fear of generating excessive family burden [24, 29]:

*… they were doing dancing and swimming*, *and we’ve just had to say look guys*, *I’m sorry*, *but we just can’t do anything*, *so nobody does anything*, *it’s just all therapy*. [28]

*My daughter would have to bring me and that would mean [her] taking time off work*.[24]

#### Biological disruption

Treatments also generated biological disruptions in terms of physical side effects such as pain, nausea, dizziness, breathlessness, fatigue, infection which were reported in over half of the included studies [3, 7, 8, 11, 26–28]. The nature, severity and frequency of physical side-effects varied substantially across the studies and were related to the type of treatment received. The PEG feeding study described the greatest range of both type and severity of symptoms. This may be due to the invasive, restrictive and technically complex nature of the intervention. However, the study on dialysis (which is also invasive, restrictive and complex) did not discuss any physical symptoms.

The extent of perceived biological disruption varied from person to person: the same treatment could generate symptoms perceived as intolerable by some and relatively minor by others:

*One participant described intubation saying*, *“If (the tube] doesn't go in right*, *they cut you up*. *You bleed*, *you're hurting*, *so on and so forth*. *Once it is in you can't talk*. *Your mouth is dry and it hurts*, *even when they take it out*. *In contrast*, *another participant*, *pointing out that he was not conscious at the time he was intubated*, *said*, *"at no time did I know that [the tube] was going in*…. *I do remember waking up after several days*…. *It did annoy me*, *but not to the point of hurting*.*"* [29]

The severity of physical side-effects did not seem to be directly associated with perceived burden; rather it was the impact that physical side effects (and indeed other types of burden) had on people’s ability engage in meaningful activities and on their personal identity that was most pertinent.

The synthesis highlighted not only how treatment burdens were experienced by patients but also the strategies patients used to minimise and manage the capability disruptions generated by treatment. Two third-order constructs were identified: “rationalised non-adherence”, strategies directed at modifying the treatment; and, “adaptive treatment work”, strategies directed at modifying the self.

#### Rationalised non-adherence

Rationalised non-adherence, which describes patients’ intentional partial or total non-adherence to treatment recommendations with the aim of minimising biographical, relational or biological disruptions, was reported in the majority of studies [7, 8, 11, 24–29]. Some patients reported ‘trial and error experiments’ with the timing or dosage of medications [11, 27]. Patients with CF and PCD [25, 26] substituted boring or difficult physiotherapy with more enjoyable sporting activities which they reframed as “treatments”. They also admitted using rationalised non-adherence as a strategy for maintaining control and feeling carefree. One woman admitted not getting the next Botox injection in the summer because she would rather tolerate the voice deficit than become breathless and not be able to go hiking [7]. Thus, rationalised non-adherence was situational and variable over time depending on the relevant competing priorities in people’s lives. Most rationalised non-adherence decisions appeared to have been taken by patients without much discussion with Health Care Professionals (HCPs) and actively concealed from them. When rationalised non-adherence was revealed, HCPs often expressed disappointment or disapproval and tried to persuade patients to adhere. This often resulted in patients feeling guilty or not understood. An exception to this was treatments offered at the end of life. In these cases HCPs often helped people to make decisions about avoiding or withdrawing invasive treatments such as dialysis or assisted ventilation[24, 29]. Clinicians working with people at end of life appeared comfortable with facilitating and sanctioning ‘rationalised non-adherence decisions’ in order to reduce BoT and maximise quality of life.

#### Adaptive treatment work

“Adaptive treatment work” describes the biographical, sentimental and relational work that patients and families engaged in as they sought to psychologically normalise treatments to their lives and their lives to the treatment. Whilst rationalised non-adherence involved changing or abstaining from treatment, adaptive treatment work involved changing how patients saw themselves or were seen by others.

Patients’ use of strategies to prevent or minimise emotional distress was common throughout these studies. We refer to this as sentimental work. Some patients sought information and reassurance from family, on-line reports, or other patients to reduce their emotional distress. Others used “mental strategies” such as distraction, social comparison and psychological preparation. Adolescents with CF [25] described a process of ‘purposeful forgetting’ to minimise the emotional impact of treatments whilst people with CKD [28] talked about the need to ‘be grown up’ about treatments, to keep the ‘desire to be normal at bay’ and develop a ‘healthy mental attitude’.

*Have a healthy mental attitude towards it … because if you let it get you down you know it could quite easily destroy you…at least you are still alive and at least there is hope*. [28]

Duration of illness seemed to play a part in enabling people to psychologically adjust to and embed treatments into lifestyles:

*I’d recommend the PEG to anyone*. *You could work with it*. *I do lots of woodwork (he made his garden gates) and gardening … it shouldn’t affect you … it’s not so bad when you’ve never been well*. *It must be very hard for people who’ve always been well*, *but I’ve been ill for 50 years*. [8]

People also engaged in biographical work, using reframing to help them maintain a valued identity. One woman adopted new crafts, allowing her to reframe how she and others saw her ‘bedbound’ lifestyle from “being lazy” to being “productive. Others framed themselves as “fighters” or “survivors” to help them bear treatment side-effects.

Relational work with family and friends was key to minimising the negative impacts of treatment on patients. This included performing treatments in secret and managing others’ expectations of their abilities.

*I don’t think people understand it (the effects of BOTOX)*. *There are really understanding people and then others*, *it’s like ‘well you talk most of the time so why can’t you do that [now]?’ … And I’ve tried to explain that the toxin wears off and so that’s a real dilemma*. [7]

Fig 2 summarises the findings, indicating how the work of treatment generates biological, biographical and relational capability disruptions and identifying how these disruptions reduce patients’ capacity resulting in adaptive work to minimise the disruptions and restore capacity.

## Discussion

### Strengths and limitations

This review is the first to explore and understand how treatments generate biological, biographical and relational disruptions in a range of conditions across the life course and illness trajectory. Moreover, we have been able to identify additional secondary treatment work required to minimise those disruptions: the combined strategies of adaptation and rationalised non-adherence. Eleven empirical studies were included, reporting on the perspectives of treatment burden from a total of 294 patients, across a range of age groups and countries. However, there are a number of limitations to our review. For instance, although we adopted a thorough and comprehensive search strategy, some relevant studies may not have been identified. We wanted to focus on gathering emergent data from patients’ own perspectives (rather than more researcher-led deductive methods) and therefore included only papers which used inductive qualitative methods suitable for generating depth data. This may have resulted in the exclusion of potentially relevant data gathered using structured postal, on-line or telephone surveys. Further, we restricted our search to English and Portuguese reports as we had no resources for translation. However, we consider our analysis to have produced an integrated model with sufficient explanatory power to explain the relationships between treatment workload, capacity, disruptions and adherence. The quality of included studies may affect the validity of our findings: although we undertook quality review of all relevant studies, in the absence of consensus on the best way to appraise qualitative research [32], and because we wanted to maximise the reach and comprehensiveness of our findings, we did not exclude on this basis. All aspects of data extraction, quality appraisal and data analysis were conducted by teams of two researchers, with a third party for disagreements. This minimised researcher bias and enhanced analysis. Framework analysis provided a robust theoretical underpinning, using existing models of treatment burden such as NPT and the cumulative complexity model to inform analytical development. We consider this an effective approach but acknowledge that a priori frameworks risk forcing data inappropriately. We sought to minimise this risk, deliberately moving from our initial deductive use of these a priori models (to fractionate and manage the data) into inductive thematic analysis specifically focussed on the perceived impact of treatment burden and the strategies employed to manage or reduce this. A further strength of this work is the inclusion of an expert patient researcher who contributed to the data analysis and writing of the paper, challenging underdeveloped conceptions and confirming the saliency of the findings to his own experiences and those of other people with chronic illness that he works with.

### Treatment generated disruptions

Over recent years great strides have been taken to clarify conceptual and operational definitions of treatment burden. To date, much of this research has focused on the burden arising from treatment workload and the impact that workload has on the maintenance of normal activity. For instance, Gallacher’s work [11, 12] has highlighted the steps people take to embed treatments into their daily lives; Eton’s work [3] has considered the impact of patient functioning on wellbeing; and Sav’s work [14, 27] has indicated that treatment burden consists of both objective elements, such as total workload and work complexity, and subjective, patient specific, elements. By synthesising the evidence across qualitative studies of treatment burden, our review has been able to expand further on these subjective elements and impacts.

Our findings support the theory proposed by May, Eton [33], by showing that there are important factors, in addition to the complexity of treatments and the time involved in enacting them. This means that treatment burden is brought about by both the workload associated with treatment, and the impacts that workload and treatment complexity have on everyday life, valued daily activities and patient identity. In this way, we have shown that treatments and their total workload cause disruptions to a person’s biological, biographical and relational capacity. Despite finding, in line with previous literature [4–6], that treatments lead to biological disruptions in the form of physical symptoms and side-effects (such as pain and nausea), our findings also indicate that it is often not the severity of symptoms that determines how burdensome treatments are: rather, that the biographical and relational disruptions arising from those symptoms and side-effects have important impacts for patients. For instance, treatments had effects on identity, interaction with others and, in many cases, were associated with negative affective states. These affective states include anxiety, fear, anger, and frustration. In some cases these symptoms were severe and debilitating, further impacting on independence, relationships with others and ultimately, adherence to treatment regimens. Whilst previous work in this field has highlighted consequences such as fatigue [8, 27] and frustration [3, 7, 8, 11, 24, 27, 30], the results of this review indicate that the psychological and biographical consequences may be more far reaching and severe than initially considered. Further research is required to explore the impact and severity of negative affect and biographical disruption arising from treatment burden, in order to investigate relationships with quality of life, treatment adherence and outcome.

### Strategies for minimising treatment disruptions: adaptation

This review also adds to the conceptual armoury of treatment burden theory by identifying two strategies for minimising treatment burden and disruptions: the first of these is adaptation. Building on previous research [1, 2, 11, 12, 31, 34] we identified three forms of adaptive work:

1. Patients engaged in their own form of *Sentimental work* to manage the negative affective states associated with treatment burden. This involved self-soothing behaviours, managing contact and interactions with friends and family, and developing other strategies to minimise distress. This builds on the work of Corbin and Strauss [1] by extending the concept of ‘sentimental work’ to work within a patient’s scope, rather than work limited to the remit of health professionals. Indeed, in a self-management care model where much of the day-to-day work of illness management is delegated to patients, sentimental work is an important part of that delegated work.
2. *Biographical work* was employed by patients to manage changes in their identity brought about by the burden of treatment. This builds on Bury’s description of illness work [2] by proposing that, in a healthcare model that has added treatment work to a patient’s total self-care workload, patients need to engage in biographical work to maintain existing or adapt to new identities caused by treatment-generated changes.
3. *Relational work* was carried out to maintain relationships. Earlier burden of treatment research, which was more focussed on the planning, doing and monitoring work of treatment [3, 11, 12], has discussed the concept of ‘treatment related relational work’. However, this concentrated on the strategic mobilisation of others to facilitate treatment. Our review extends this research by addressing the work that patients do to minimise the impact of treatment on valued relationships with self and others. In this way, patients use relational work to draw on a wider workforce [11, 12, 34] but also to minimise relational disruption and maximise the work output from that workforce by maintaining optimal, productive and agreeable relationships.

### Strategies for minimising treatment disruptions: rationalised non-adherence

We identified a second strategy to minimise treatment burden: rationalised non-adherence. We found that non-adherence was often not an arbitrary act or sign of personal moral failure as it is sometimes viewed [35, 36] but a rationalised process, used actively and mindfully, as a way of minimising the burden of treatment. Karamanidou, Weinman [28] discusses a similar phenomenon in patients undergoing haemodialysis, which they called ‘active non-adherence’. They attributed this non-adherence to beliefs about the importance (or not) of treatments, however, we noted non-adherence associated with rationalised decisions based on a desire to minimise the disruptions associated with treatment. As a result, we have termed this strategy ‘rationalised non-adherence’. Rationalised non-adherence occurred when patients, having appraised the impact of treatment, deliberately decided to cease, modify or reduce their treatment regime.

We identified two distinct forms of rationalised non-adherence. We found that in studies which included populations approaching the end of life, non-adherence decisions were frequently sanctioned and supported by clinicians [24, 29]. Indeed, recent policy and practice initiatives recommend open discussion about end of life treatment decisions and include directives to support and endorse patients’ decisions, even where these are decisions not to treat [37–39]. This is in contrast to how non-adherence was discussed in studies exploring less critical situations [14, 25–28]. We found that rationalised non-adherence at other points in the illness trajectory or life course was a ‘secret-act’ that must be hidden from others. Such (non)-treatment decisions were undertaken covertly, without the knowledge, approval or guidance of professionals. This suggests a lack of concordance between patients’ and clinicians’ perspectives, which according to our data, results in feelings of guilt. It may also affect patient outcome, because important information is withheld that could affect clinical-reasoning and future treatment recommendations. Adopting some of the principles of the palliative care approach would facilitate open discussion about the impact of treatment burdens and disruption on adherence to treatment, thus providing healthcare professionals with an opportunity to consider the balance between treatment burden, adaptation and adherence. Links between treatment burden and treatment adherence have also been discussed by May, Montori [40] in their seminal text on Minimally Disruptive Medicine. They propose that treatment burden can lead to structurally induced non-compliance as a result of increasingly complex and abundant treatment regimens. In this way, structurally induced non-compliance might be viewed as an overarching term linking treatment burden with adherence. Our analysis allowed us to explore these links in greater depth and we have shown that rather than being solely a response to treatment overload, non-adherence is often an adaptive and rationalised process employed to minimise treatment disruption.

### Future developments for BoT research

Whilst we have been able to partially explain the link between treatment burden, disruption and adherence, we have been unable to explore the impact of this non-adherence on relationships with healthcare professionals and family. The data suggest that rationalised non-adherence necessitates further work in the form of concealment, persuasion, and the recruitment of allies among family and health professionals. However, further empirical research is required to explore this work in more depth; to consider what this work entails, the severity of its consequences for relationships with others and its impact on outcomes.

In addition to extending our understanding of the burden of treatment by contributing to a body of work in the field, this review also builds on and adds to recent work on how capacity is expressed by patients. May, Eton [33] suggest that in order for functional performance (the potential to do the treatment work that needs to be done), patients must mobilise resources from their social capital and secure the cooperation of others in their formal and informal social network. Doing so is said to allow patients to develop structural resilience. In other words, patients must adapt in order to absorb, embed and minimise treatment related burden and disruption. This adaptation or ability to absorb adversity is an expression of capacity. Our findings support this view by identifying biographical, relational and biological capacity, and outlining a number of strategies patients use to adapt to disruptions to these components of their capacity. However, we note that these strategies involve work that can generate further burdens and disruptions. This may explain why we also identified that in some cases, functional performance is deliberately sacrificed for quality of life: rationalised non-adherence means ceasing or modifying some of the work of treatment in order to minimise disruption without the additional work of adaptation. Of course, rationalised non-adherence is still a form of adaptation, albeit one of circumvention.

## Conclusions

This framework synthesis makes a novel contribution to our understanding of treatment burden. Using evidence from the patient’s perspective we found that treatment burden is experienced as biological, biographical and relational disruptions. Patients minimise these disruptions through adaptation and rationalised non-adherence. Whilst rationalised non-adherence is supported by HCPs at end-of-life; at other times it is a ‘secret-act’ that can generate guilt, disrupt relationships with HCPs and family, and ultimately reduce health outcomes. Future work on burden of treatment should consider both treatment workload and treatment disruption to fully account for the consequences as well as experiences of treatments. Clinicians should engage patients in conversations that allow them to acknowledge treatment burden and discuss adherence difficulties without fear of judgement so that appropriate modifications can be made to ensure minimally disruptive treatments [33, 40]. Our findings suggest that in order to be minimally disruptive, treatments not only have to have a low workload (both duration and complexity), they also have to cause minimal disruption to people’s biographical, relational biological capacity.

## Supporting Information

S1 PRISMA Checklist PRISMA Checklist identifying how and where each element of the PRISMA process has been addressed in this paper. (PDF)
